# Blockade of myeloid differentiation protein 2 prevents obesity‐induced inflammation and nephropathy

**DOI:** 10.1111/jcmm.13287

**Published:** 2017-08-02

**Authors:** Qilu Fang, Lintao Wang, Daona Yang, Xiong Chen, Xiaoou Shan, Yali Zhang, Hazel Lum, Jingying Wang, Peng Zhong, Guang Liang, Yi Wang

**Affiliations:** ^1^ Chemical Biology Research Center School of Pharmaceutical Sciences Wenzhou Medical University Wenzhou Zhejiang China; ^2^ Affiliated Cangnan Hospital of Wenzhou Medical University Wenzhou Zhejiang China; ^3^ Department of Endocrinology The First Affiliated Hospital of Wenzhou Medical University Wenzhou Medical University Wenzhou Zhejiang China; ^4^ Department of Pediatrics The Second Affiliated Hospital of Wenzhou Medical University Wenzhou Zhejiang 325000 China

**Keywords:** obesity, renal injury, inflammation, myeloid differentiation 2, free fatty acid

## Abstract

Obesity is a major and independent risk factor of kidney diseases. The pathogenic mechanisms of obesity‐associated renal injury are recognized to at least involve a lipid‐rich and pro‐inflammatory state of the renal tissues, but specific mechanisms establishing causal relation remain unknown. Saturated fatty acids are elevated in obesity, and known to induce chronic inflammation in kidneys. Myeloid differentiation protein 2 (MD2) is an important protein in lipopolysaccharide‐induced innate immunity response and inflammation. We suggested that obesity‐associated renal injury is regulated by MD2 thereby driving an inflammatory renal injury. The used three mouse models for *in vivo* study: MD2 knockout mice (KO) maintained on high fat diet (HFD), wild‐type mice on HFD plus L6H21, a specific MD2 inhibitor and KO mice given palmitic acid (PA) by IV injection. The *in vitro* studies were carried out in cultured renal tubular epithelial cells, mouse mesangial cells and primary macrophages, respectively. The HFD mice presented with increased hyperlipidemia, serum creatinine and proteinuria. Renal tissue from HFD mice had increased fibrosis, inflammatory cytokines, macrophage infiltration, and activation of NF‐κB and MAPKs. This HFD‐induced renal injury profile was not observed in KO mice or L6H21‐treated mice. Mice given PA mimmicked the HFD‐induced renal injury profiles, which were prevented by MD2 knockout. The *in vitro* data further confirmed MD2 mediates PA‐induced inflammation. MD2 is causally related with obesity‐associated renal inflammatory injury. We believe that MD2 is an attractive target for future therapeutic strategies in obesity‐associated kidney diseases.

## Introduction

In recent years, epidemiological and clinical studies have linked obesity to the development and progression of kidney injury. Obesity is considered an important factor for developing end‐stage renal disease, behind only to proteinuria 1, 2, 3. Obesity‐associated kidney injury in human subjects is characterized by structural remodelling of the kidney tissue and includes tubular atrophy, interstitial fibrosis, arterial sclerosis and glomerulomegaly 2, 4. While obesity is clearly related to kidney injury, the pathophysiological mechanisms are far from clear.

A persistent and crucial component of obesity‐related tissue injury is chronic inflammation resulting from the production of pro‐inflammatory molecules 5, 6, 7, 8, 9 and macrophage infiltration 10, 11. In the kidneys of obese animal models, preventing inflammation protects against renal dysfunction and tissue remodelling 9, 11. A major driver of inflammation in obesity is elevated levels of saturated free fatty acids (SFAs) 12, 13. SFAs including lauric acid (C12:0), PA (C16:0) and stearic acid (C18:0) are pro‐inflammatory lipid compounds known to activate inflammatory signalling in a wide range of cells 14, 15, 16, 17, 18. PA is the most abundant SFA in blood, and the most studied in the pathogenesis of the metabolic syndrome, insulin resistance and vascular disorders 8, 13, 14, 19, 20. We 11 and others 8, 16 have shown that PA is a potent stimulus for the production of cytokines, adhesion molecules, as well as activation of nuclear factor‐κB (NF‐κB).

Recent studies have shown that elevated SFAs may initiate inflammatory injury through activating toll‐like receptor 4 (TLR4) 21. TLR4 is a critical pattern recognition receptor and is well known for recognizing lipopolysaccharide (LPS), a component of Gram‐negative bacteria 22. The LPS response requires a mediating protein called MD2. Upon binding to LPS, the LPS‐MD2‐TLR4 complex forms and activates downstream pro‐inflammatory signalling cascades, leading to the activation of NF‐κB, activating protein‐1 (AP‐1) and mitogen‐activated protein kinase (MAPK) 23, and then induces cytokines such as tumour necrosis factor‐α (TNF‐α), interleukin‐ (IL) 1β, IL‐6 and interferon‐γ (IFN‐γ) 15, 24, 25, 26, 27.

MD2 plays an important role in LPS‐induced innate immunity and inflammation. However, it is not known whether MD2 is required in SFA/obesity‐induced inflammation. In this study, we tested the causal relation of MD2 in renal inflammatory injury in the HFD‐induced model of obesity, PA‐challenged mice, and cultured renal cells. We demonstrate that MD2 is an important regulator of HFD‐ and SFA‐induced renal inflammatory injury.

## Materials and methods

PA and bovine serum albumin (BSA) were purchased from Sigma‐Aldrich (St. Louis, MO, USA). MD2 neutralizing antibody (anti‐MD2) was purchased from InvivoGen (San Diego, CA, USA). Compound L6H21 was prepared with a purity of 99.2% as described in our previous study 28, 29. L6H21 were dissolved in DMSO for *in vitro* experiments and in 1% sodium carboxyl methyl cellulose (CMC‐Na) for *in vivo* experiments. Antibodies for GAPDH, p38, p‐p38, JNK and p‐JNK were purchased from Cell Signaling (Danvers, MA, USA). Antibodies for IκB‐α, p‐ IκB‐α, MD2, ERK, p‐ERK, TGF‐β1, Collagen IV, NF‐κB p65, VCAM‐1, Lamin B, CD68, MCP‐1, TLR4 and CD68 were purchased from Santa Cruz Biotechnology (Santa Cruz, CA, USA). Secondary antibodies were also obtained from Santa Cruz. Mesangial cells (SV40 MES 13, ATCC‐CRL‐1927) and tubular epithelial cells (NRK‐52E, ATCC‐CRL‐1571) were obtained from American Type Culture Collection (ATCC, Manassas, VA, USA). The cells were maintained at 37°C under a humidified 5% CO2 in Dulbecco's modified Eagle's medium (DMEM) (Gibco, Eggenstein, Germany) containing 5.5 mM D‐glucose (low glucose, LG) supplemented with 10% FBS (Gibco), 100 U/ml penicillin and 100 U/ml streptomycin.

### Animal studies

Male C57BL/6 mice (8 weeks old, weighing 18–20 g) were purchased from SLAC Laboratory Animal Center (Shanghai, China). Male MD2^−/−^ mice (B6.129P2‐Ly96 KO) on a C57BL/6 background were purchased from Riken BioResource Center (Tsukuba, Ibaraki, Japan). All animal care and experimental procedures were approved by the Wenzhou Medical University Animal Policy and Welfare Committee.

#### HFD model of obesity

Male wild‐type (C57BL/6) and MD2^−\−^ (KO) mice were fed a HFD or normal control diet (ctrol) for 4 months. Body weights were recorded weekly and at the end of the 4‐month study. Normal control diet was purchased from MediScience Diets Co. LTD, Yangzhou, China, containing 10 kcal.% fat, 20 kcal.% protein and 70 kcal.% carbohydrate (Cat. #MD12031); HFD was purchased from the same company (Cat. #MD12033) containing 60 kcal.% fat, 20 kcal.% protein and 20 kcal.% carbohydrate. The role of MD2 was also examined in C57BL/6 mice treated with MD2 inhibitor L6H21. For these studies, C57BL/6 mice on control diet or HFD (2‐month duration) were treated with 20 mg/kg of L6H21 (HFD‐L6H21) or 1% CMC‐Na solution (control) once every 2 days by oral gavage for 2 months, while maintained on their respective diets. An additional group of mice was administered with curcumin (50 mg/kg) as positive control.

#### PA challenge model

Male C57BL/6 and MD2^−/−^ mice were injected with 300 μl of PA (5 mM), normal saline control (NS) or BSA vehicle control by tail vein twice daily for 2 weeks. At the end of treatment period, mice were killed, and urine, blood and kidney tissues were collected. Kidney tissues were weighed and fixed in 4% paraformaldehyde for morphological analysis or snap‐frozen in liquid nitrogen for gene and protein expression analyses. Serum and urine samples were stored in −80°C.

### Additional methods

Routine methods including biochemical measurement, cell culture, isolation and culture of primary macrophages from rats or mice, macrophage adhesion assay, Western blotting, immunohistochemistry, real‐time qPCR and primers used for real‐time qPCR assay (Table S1) are described in details in the Supplementary File.

### Statistical analysis

Data were presented as means ± S.E.M. Differences were detected by Student's *t*‐test or anova multiple comparisons as appropriate using GraphPad Pro (GraphPad, San Diego, CA, USA). *P* < 0.05 was considered significant.

## Results

### MD2 deficiency prevents renal inflammation and injuries in the HFD‐induced model of obesity

**Figure 1:**
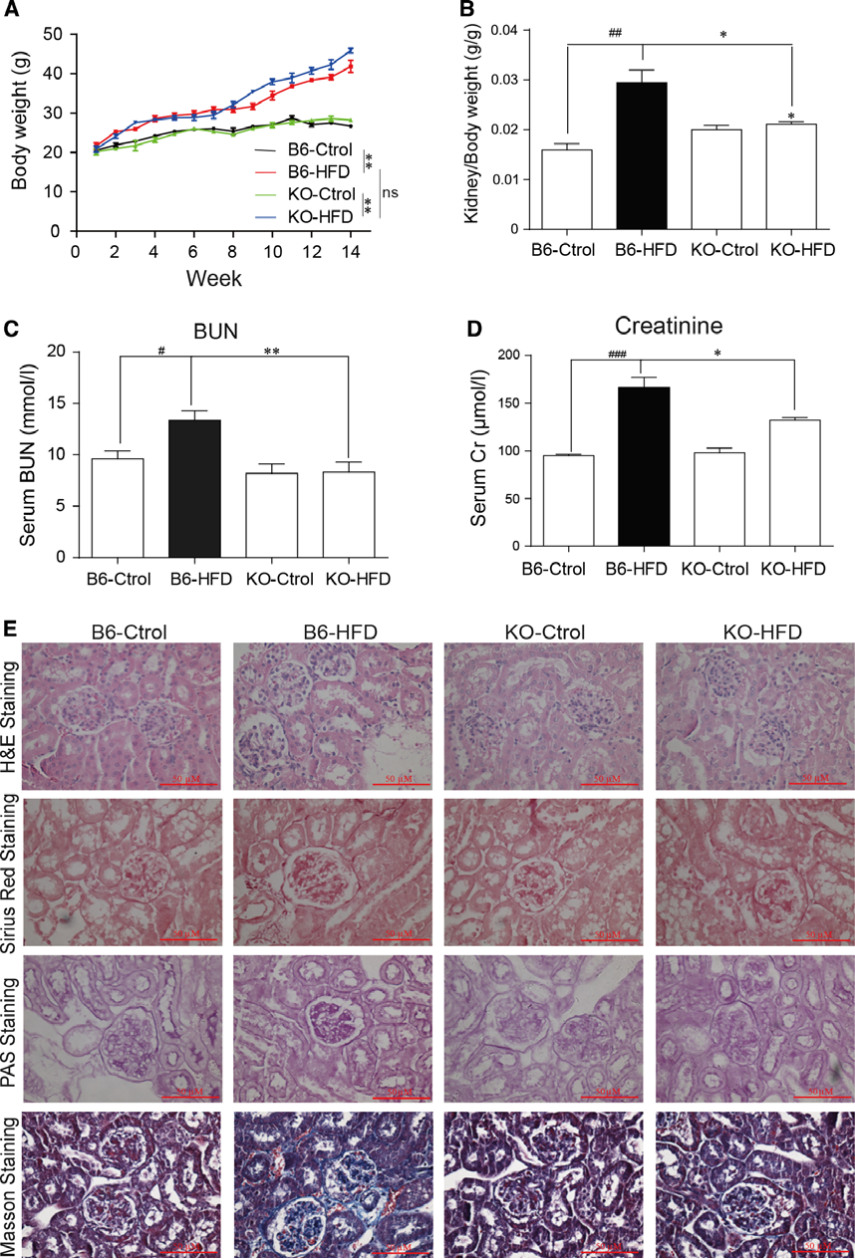
MD2 knockout mice are protected against high fat diet‐induced renal dysfunction. Wild‐type (B6) and MD2^−/−^ (KO) mice were fed a HFD or control diet (Ctrol) for 4 months, and kidney tissues were evaluated for inflammatory injury. (**A**) Body weights of mice fed Ctrol and HFD. (**B**) Kidney weights normalized to body weight. (**C**) Serum BUN levels. (**D**) Serum creatinine levels. (**E**) Representative microscopic images showing H&E, Sirius Red (red colour), PAS (purple colour) and Masson's trichrome (blue colour) staining. The quantitative data for 1E were shown in the Figure S3. [means ± S.E.M.; *n* = 7; *P < 0.05, **P < 0.01, B6‐HFD *versus* KO‐HFD; #*P* < 0.05, ##*P* < 0.01, and ###*P* < 0.001, B6‐Ctrol versus B6‐HFD].

To study the potential role of MD2 in obesity‐associated renal injury, we utilized the HFD‐induced model of obesity 30, 31. We first examined whether the expression and activity of MD2 are altered in the kidney tissues of HFD‐fed mice. Our results show that 4 months of HFD feeding induced MD2 expression and MD2/TLR4 complex formation in the kidney tissues (Fig. S1). We then subjected MD2^−/−^ mice to the same HFD regimen for 4 months. Maintaining wild‐type C57BL/6 (B6) and MD2^−\−^ (KO) mice on HFD resulted in an increased weight gain compared to mice fed a control diet (Fig. 1A). However, analysis of kidney tissues revealed that only B6 mice had increased kidney to body weight ratio (KW/BW) but not MD2‐KO mice (Fig. 1B). B6‐HFD mice also presented with hyperlipidemia as indicated by increased circulating low‐density lipoproteins (LDL), triglycerides (TG) and total cholesterol (TCH) (Fig. S2). KO‐HFD mice showed similar increases in the lipids except for TG, which did not show any alteration compared to KO mice on control diet (Fig. S2B).

Analysis of renal function parameters showed that HFD feeding increased serum levels of creatinine and blood urea nitrogen (BUN) in B6 mice (Fig. 1C and D) compared to mice on control diet. However, BUN and creatinine did not show any alteration in the KO‐HFD mice indicating preserved kidney function. These results indicate preserved renal function in MD2^−/−^ mice fed a HFD.

**Figure 2:**
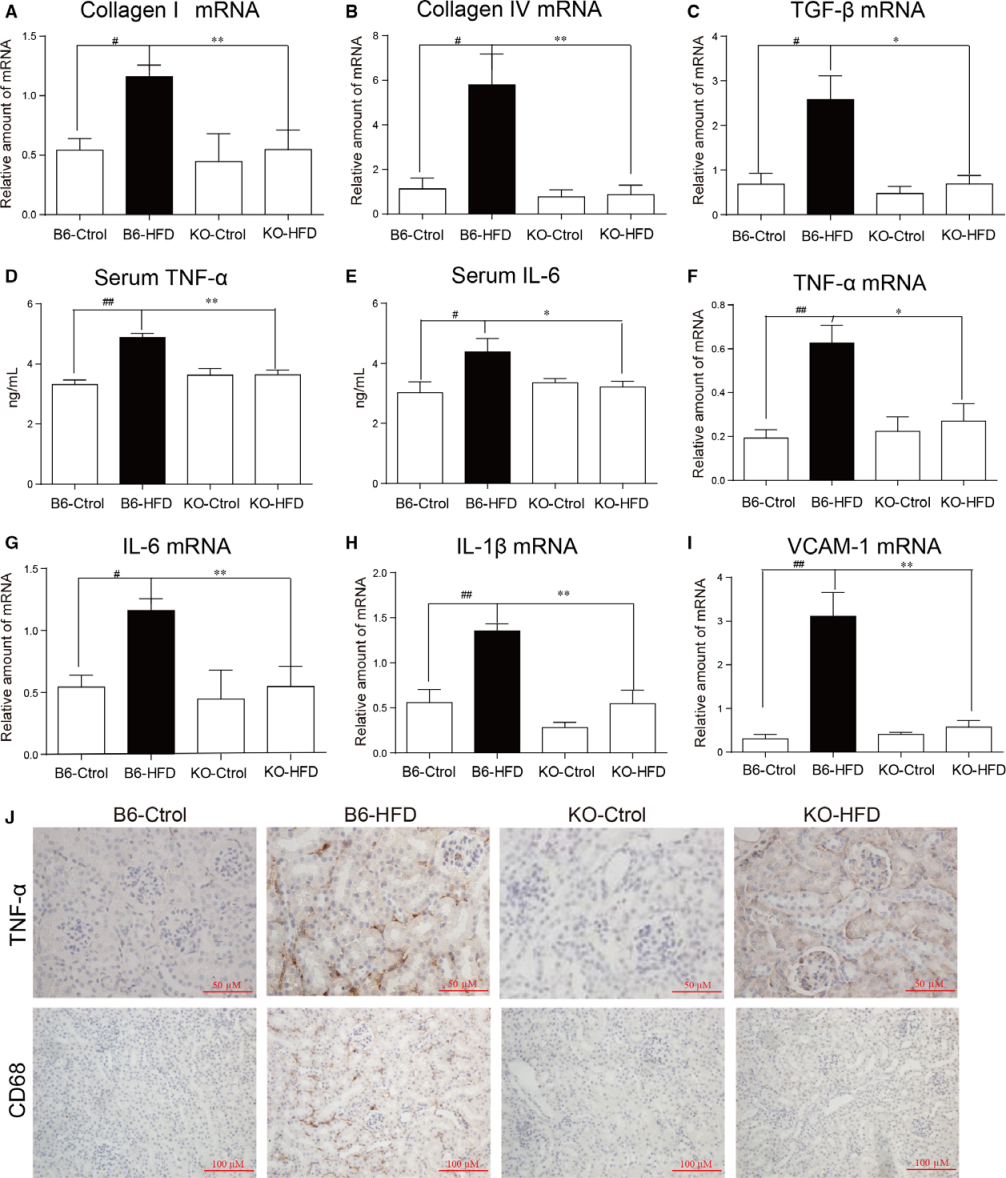
Lack of renal tissue inflammation in MD2 knockout mice maintained on high fat diet. (**A**‐**C**) Real‐time quantitative PCR analysis of Collagen I (**A**), Collagen IV (**B**) and TGF‐β1 (**C**) mRNA levels in kidney tissues of wild‐type B6 and MD2 KO mice. (**D**,** E**) Serum levels of TNF‐α (**D**) and IL‐6 (**E**) in kidney tissue homogenates. (**F**‐**I**) PCR analysis of inflammatory mediators showing TNF‐α (**F**), IL‐6 (**G**), IL‐1β (**H**) and VCAM‐1 (**I**) mRNA levels. (**J**) Representative microscopic images showing TNF‐α and CD68 immunohistochemistry (brown colour). An amplified image and a quantitative result for TNF‐α staining were shown in the Figure S4. [mRNA levels normalized to β‐actin; mean ± S.E.M.; *n* = 7/8; *P < 0.05, **P < 0.01, and ***P < 0.001, B6‐HFD versus KO‐HFD; #*P* < 0.05, ##*P* < 0.01, and ###*P* < 0.001, B6‐Ctrol versus B6‐HFD].

We next examined structural alterations in the kidney tissues of HFD‐fed mice using immunohistological staining (Fig. 1E and Fig. S3). Kidney tissues from B6‐HFD mice showed increased deposition of connective tissue within glomeruli, interstitial‐tubular region and in the Bowman's capsule as detected by Masson's trichrome and Sirius red staining. Periodic Acid‐Schiff (PAS), another commonly used connective tissue stain, showed similar results. These features are consistent with our previous study 11 and are indicative of increased mesangial matrix expansion seen in obesity‐associated renal injury 2, 10. Interestingly, this tissue remodelling activity was not evident in the KO‐HFD mice (Fig. 1E). Furthermore, mRNA levels of collagen I and collagen IV, and transforming growth factor‐β1 (TGF‐β1) were increased in B6‐HFD mice but showed no alteration in the KO‐HFD mice (Fig. 2A–C).

Glomerulopathy in obesity is associated with an inflammatory state of the kidney 3. We have recently shown increased production of pro‐inflammatory factors in the kidney tissues of mice maintained on HFD 11. Therefore, we investigated whether preserved renal function and inhibited tissue remodelling observed in MD2 KO mice on HFD is associated with reduced inflammatory activity. Indeed, our results show no alteration in serum levels of tumour necrosis factor‐α (TNF‐α) and interleukin‐6 (IL‐6) in KO‐HFD mice compared to KO mice fed the control diet (Fig. 2D and E). mRNA levels of cytokines in KO‐HFD, including TNF‐α, IL‐6, IL‐1β and adhesion molecule, vascular cell adhesion molecule‐1 (VCAM‐1) also remained at levels comparable to KO mice on control diet (Fig. 2F–I). Immunohistochemical staining showed increased TNF‐α and macrophage marker CD68 immunoreactivity in the kidney tissues of the B6‐HFD mice (Fig. 2J and Fig. S4). The staining was dramatically lower in the KO‐HFD mice. As these cytokines and adhesion molecule are elevated only in the B6 mice on HFD, the results clearly show inhibited inflammatory activity in the MD2 deficient mice.

### Inhibiting MD2 by small‐molecule inhibitor prevents development of kidney dysfunction in HFD‐induced model of obesity

Our studies in MD2 KO mice show that MD2 activity is involved in HFD‐induced obesity and associated renal dysfunction. To build on these results, we tested whether we can prevent obesity‐associated renal dysfunction using another approach to inhibit MD2. We used a small‐molecule inhibitor of MD2, L6H21, which specifically targets MD2 through direct binding 32. Curcumin is a well‐known natural product with anti‐inflammatory activity and exhibits anti‐inflammatory activity and inhibits HFD‐induced renal inflammation and injuries 33, 34, 35. Here, curcumin was used as a positive comparison. We performed similar studies with L6H21 as with the MD2 KO mice. For this study, MD2 inhibition by L6H21 administration was started after 2‐month HFD feeding. We also tested the MD2 expression in kidneys of mice fed by HFD for 2 months. The Figure S5 showed that 2 months of HFD feeding already induced MD2 overexpression and MD2/TLR4 complex formation in mouse kidneys.

**Figure 3:**
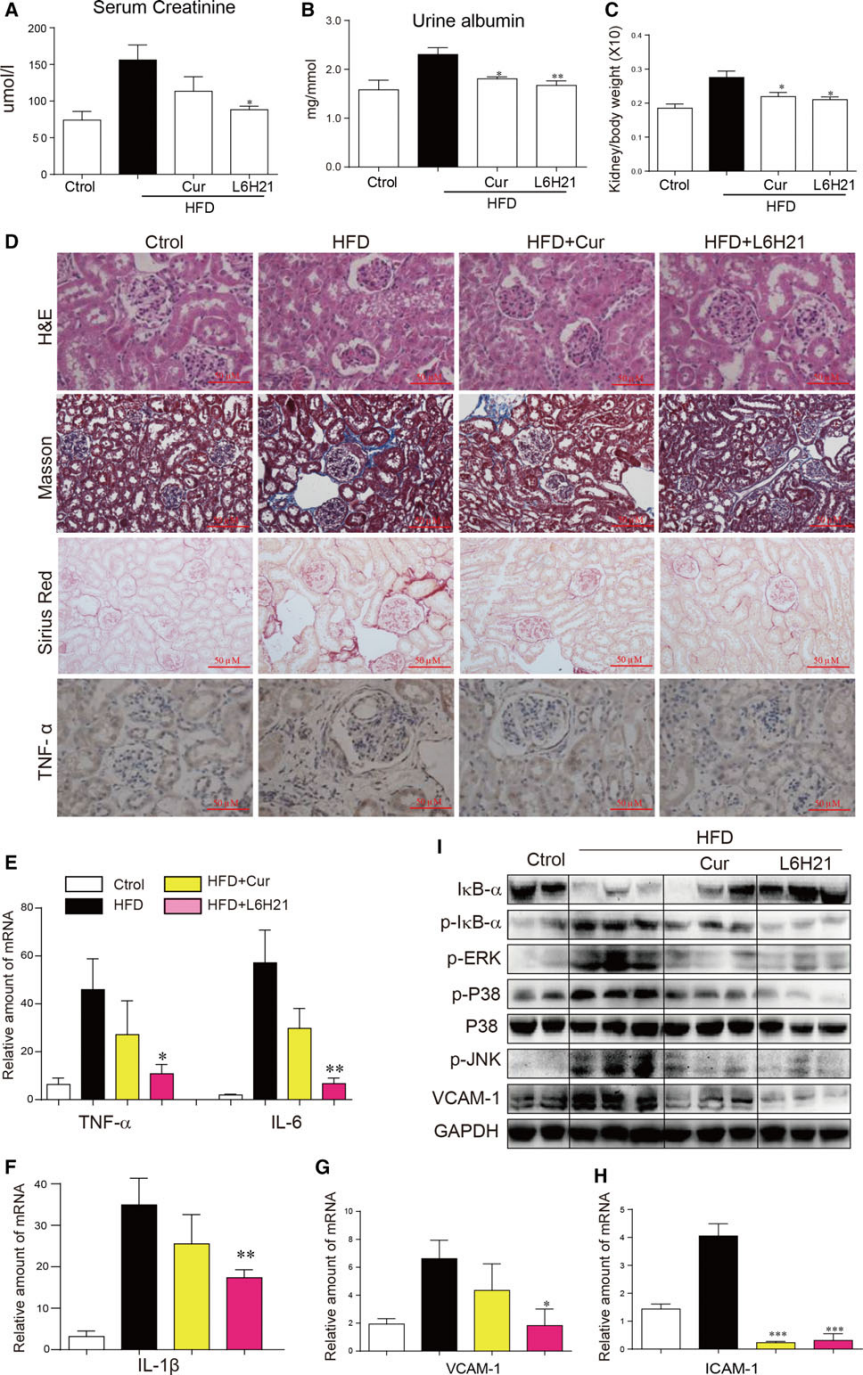
MD2 inhibition by L6H21 protects against high fat diet‐induced kidney remodelling and inflammation. C57BL/6 mice were fed a HFD or control diet (ctrol) for 2 months. Mice were then treated with L6H21 every 2 days for 2 months and kidney tissues were evaluated. (**A**) Serum creatinine levels in kidney tissue homogenates. (**B**) Urine albumin levels after 2 months of treatment with L6H21. (**C**) Kidney weights normalized to body weight. (**D**) Representative microscopic images of H&E, Masson's trichrome stain (blue colour), Sirius Red stain (red colour) and immunohistochemical staining for TNF‐α (brown colour). The quantitative data for 1E were shown in the Figure S7. (**E**‐**H**) PCR analysis of inflammatory genes showing TNF‐α and IL‐6 (**E**), IL‐1β (**F**), VCAM‐1 (**G**) and ICAM‐1 (**H**) mRNA levels in kidney tissues of B6 mice. (**I**) Representative Western blot analysis of inflammatory signalling pathway activation. [Cur = curcumin/positive control; mRNA levels normalized to β‐actin; means ± S.E.M.; *n* = 7/8 in four groups; * *P <* 0.05, ** *P <* 0.01, and ***P < 0.001 versus HFD].

First, we assessed the effect of L6H21 on hyperlipidemia in B6 mice on HFD. Our results show that L6H21 did not affect the levels of LDL, TG or TCH in B6 mice fed a HFD (Fig. S6). However, parameters of HFD‐associated renal dysfunction including increased serum creatinine (Fig. 3A) and urine albumin (Fig. 3B) were prevented by L6H21. L6H21 also normalized KW/BW ratio (Fig. 3C) and reduced measures of connective tissue deposition in mice fed with HFD (Fig. 3D and Fig. S7). These results show that MD2 inhibition ameliorated HFD‐induced structural and functional alterations in the kidney.

We next assessed indices of inflammation in the kidney tissues of B6 mice treated with L6H21. Anti‐CD68 immunofluorescence staining analysis showed that L6H21 blocked HFD‐induced macrophage infiltration in mouse kidney (Fig. S8). TNF‐α immunoreactivity in renal tissues of B6 mice maintained on HFD and treated with L6H21 was comparable to mice fed normal control diet (Fig. 3D and Fig. S7). This result correlated with mRNA levels of TNF‐α (Fig. 3E) and other cytokines including IL‐6 and IL‐1β (Fig. 3E–F). Similarly, we found that mRNA and protein levels of adhesion molecules VCAM‐1 and ICAM‐1 and chemokine MCP‐1 in HFD mice treated with L6H21 were comparable to mice on control diet (Fig. 3G–I and Fig. S9). These results indicate that L6H21 prevented HFD‐induced renal dysfunction possibly through dampening inflammatory activity. Therefore, we examined the activity of signalling proteins involved in the expression of pro‐inflammatory molecules. Studies have shown that MAPK and NF‐κB are downstream of MD2/TLR4 complex and these signalling pathways are also the predominant ones activated in renal tissue in mice on HFD 9, 11. We found reduced phosphorylation and degradation of IκB‐α in L6H21‐treated mice fed a HFD compared to HFD alone group (Fig. 3I). Additionally, L6H21 prevented the HFD‐associated increased phosphorylation of ERK, p38 and JNK in renal tissues (Fig. 3I).

### PA induces MD2‐dependent renal inflammatory injury *in vivo*

**Figure 4:**
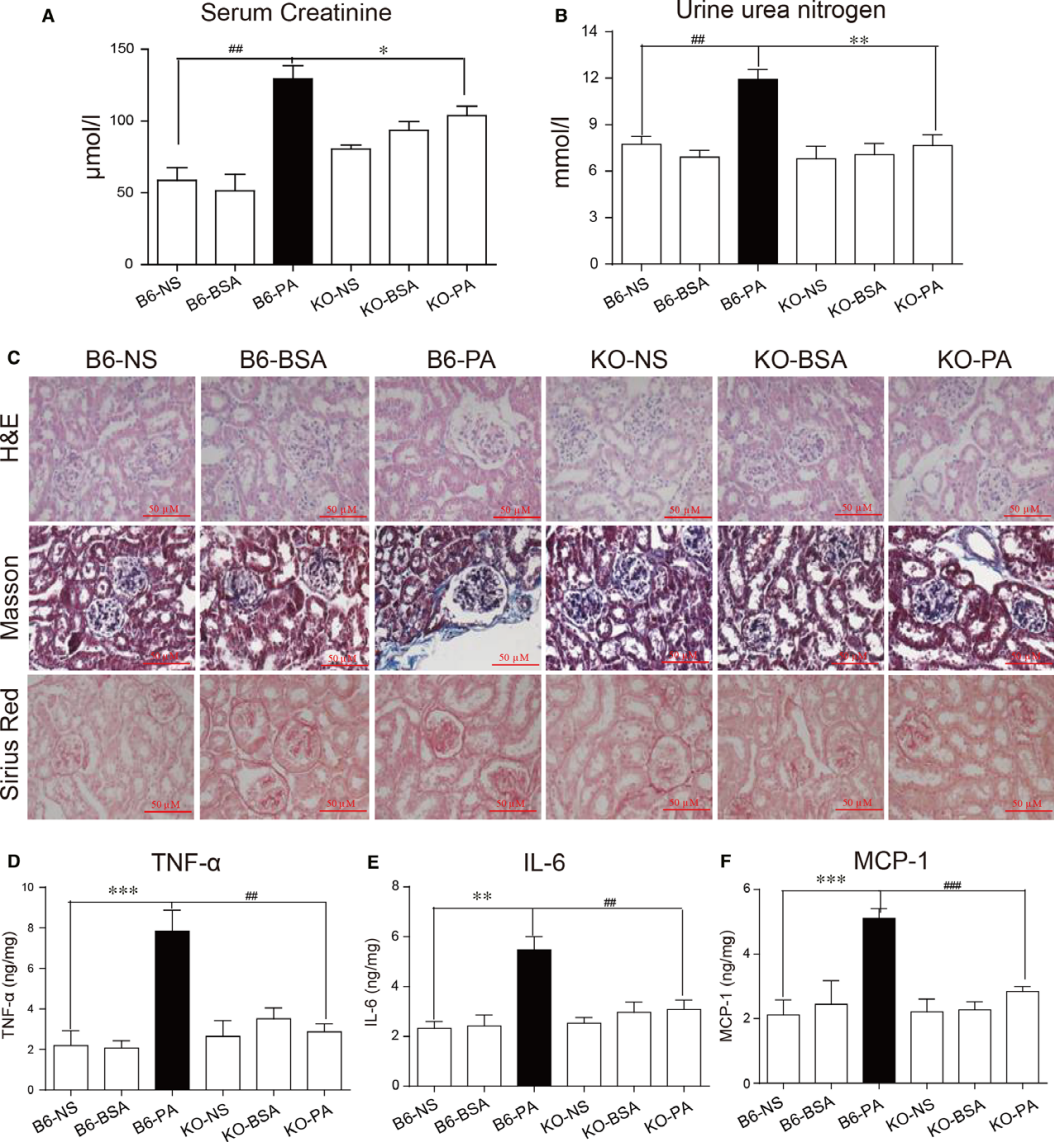
Palmitic acid induces MD2‐dependent renal dysfunction and tissue remodelling *in vivo*. Wild‐type (B6) and MD2^−/−^ mice (KO) were challenged with palmitic acid (PA) twice daily for 2 weeks and urine samples and kidney tissues collected for analysis. (**A**) Serum creatinine levels in mice challenged with PA. (**B**) Urine urea nitrogen levels. (**C**) Representative histochemical assessment of kidney tissue morphology by H&E, and fibrosis by Masson's trichrome and Sirius Red staining. The quantitative data for 1E were shown in the Figure S12. (**D‐F**) Pro‐inflammatory cytokines in kidney tissues of PA‐challenged mice showing TNF‐α (**D**), IL‐6 (**E**) and chemotactic factor MCP‐1 (**F**). (mRNA levels normalized to β‐actin; mean ± S.E.M.; *n* = 8; **P < 0.01, and ***P < 0.001, KO‐PA versus B6‐PA; ##*P* < 0.01, and ###*P* < 0.001, B6‐NS versus B6‐PA).

Elevated fatty acids in the HFD‐fed mice may have mediated inflammatory activity in the kidney tissues leading to tissue structural and functional alterations. We measured local level of free fatty acids (FFAs) in kidney tissues of mice fed a HFD and show elevated levels (Fig. S10). To explore a potential mechanistic role, we challenged wild‐type B6 and MD2 KO mice with PA, the most predominant saturated FFA which plays a detrimental role in tissues when excessed 14, 15, 16, 17, 18. Here, a significant limitation in Figure S10 is that we did not directly measure the PA (or total saturated FFAs) level in kidneys from control and HFD mice. As expected, we found that B6 mice challenged with PA developed kidney weight increase (Fig. S11) and renal dysfunction as indicated by elevated serum creatinine and urine urea nitrogen (Fig. 4A and B). Microscopic examination revealed increased connective tissue deposition in PA‐challenged B6 mice (Fig. 4C and Fig. S12). Both renal dysfunction measures and tissue remodelling were not observed in the MD2 KO mice treated with PA (Fig. 4A–C and Fig. S12). Furthermore, KO mice treated with PA presented with levels of TGF‐β1 and collagen I mRNA that were comparable to that of control (Fig. S13A and B).

We next examined pro‐inflammatory factor expression in PA‐challenged mice and show increased TNF‐α, IL‐6 and MCP‐1 protein in B6 mice (Fig. 4D–F). However, this induction was not observed in the KO mice. PA also induced renal tissue mRNA expression of cytokines and adhesion molecules in B6 mice but not the MD2 KO mice (Fig. S13C–G). The up‐regulation of adhesion molecules in the renal tissue suggests recruitment of infiltrating leucocytes. We found TNF‐α to demark the interstitial‐tubular sites of the kidney and as expected, CD68 was also detected in mice treated with PA (Fig. S13H). Importantly, PA‐induced TNF‐α and CD68 immunoreactivity was reduced in KO mice (Figs S13H and S14).

### Inflammatory locus of tubular epithelial cells and mesangial cells is MD2‐dependent

**Figure 5:**
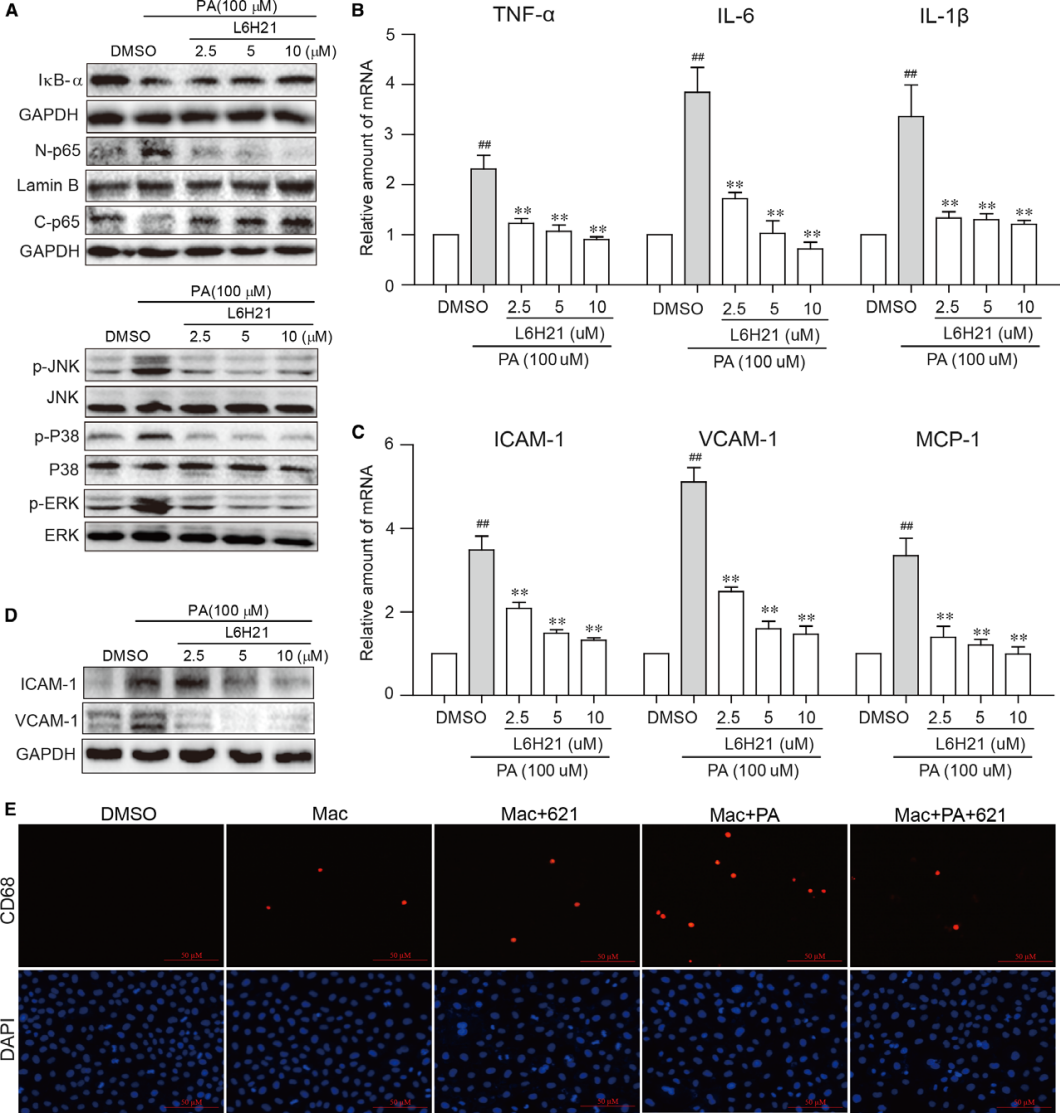
Palmitic acid activates MD2‐dependent inflammatory activity in NRK52E cells. Rat kidney epithelial cells NRK52E were exposed to PA for indicated times with or without 1 hrs pre‐treatment with L6H21. (**A**) Representative Western blot analysis of NFκB and MAPK activation by PA showing IκB‐α, nuclear p65 and cytosolic p65 (following nuclear protein extraction), p‐ERK, p‐JNK and p‐p38 in NRK52E cells (30 min. PA exposure). (**B**) qPCR analysis of inflammatory cytokines showing TNF‐α, IL‐6 and IL‐1β mRNA levels in NRK52E cells stimulated with PA for 6 hrs. (**C**) mRNA expression of adhesion molecules (ICAM‐1 and VCAM‐1) and MCP‐1 in NRK52E cells stimulated with PA for 6 hrs. (**D**) Representative Western blot of ICAM‐1 and VCAM‐1 in NRK52E cells stimulated with PA for 12 hrs. (**E**) Rat primary macrophage adhesion to PA‐treated NRK52E cells as assessed through CD68 staining (Red). [mRNA normalized to β‐actin; means ± S.E.M.; *n* = 3; #*P* < 0.05, ##*P* < 0.01, and ###*P* < 0.001 versus DMSO.]

To gain insight into target cells of HFD‐ and PA‐induced renal damage, we investigated renal tubular epithelial cells (NRK52E) and renal mesangial cells (SV40). We stimulated NRK52E cells with 100 μM PA and assessed the activation of signalling proteins in the MD2/TLR4 pathway. PA‐activated NF‐κB as shown by increased IκB‐α degradation and NF‐kB p65 translocation from the cytosol to nucleus. In addition, PA activated the three arms of the MAPK pathway (ERK, JNK and p38) (Fig. 5A). Activation of these pathways was associated with increased mRNA levels of TNF‐α, IL‐6 and IL‐1β (Fig. 5B). PA‐induced signalling protein activation and cytokine up‐regulation were effectively prevented by L6H21 (Fig. 5A and B). Additionally, PA‐activated NRK52E cells presented with increased mRNA expression of adhesion molecules (ICAM‐1 and VCAM‐1) and chemoattractant MCP‐1 (Fig. 5C), and the corresponding proteins (Fig. 5D). We also found that PA‐stimulated NRK52E cells were more adhesive to rat primary macrophages (Fig. 5E). Pre‐treatment of NRK52E cells with L6H21 significantly inhibited adhesion molecule up‐regulation in a dose‐dependent manner and remarkably prevented macrophage adhesion in NRK52E cells (Fig. 5C–E). Similar results were observed in mouse SV40 mesangial cells (Fig. S15). These results indicate that PA induces MD2‐dependent inflammation in renal tubular cells and mesangial cells.

## Discussion

Obesity is associated with the development of kidney disease, and there is an urgent need to understand the pathogenic drivers of injury progression. In our study, we have used established mouse models of obesity to test a novel mechanism centred on inflammatory injury. Our results showed that kidneys in obese mice express more MD2 protein. Renal tissue inflammation, tissue remodelling and functional alterations did not occur in HFD‐fed mice when MD2 gene was disrupted or pharmacologically inhibited. We also found that PA mimicked HFD and PA‐induced alterations were also inhibited by MD2. Our study suggests that MD2 is essential for the development and the progression of renal inflammatory injury in obesity.

Mice fed a HFD presented with a greater KW/BW ratio than mice fed the standard chow. This ratio was maintained despite a steady gain of body weight. Importantly, the KW/BW ratio in the KO‐HFD mice was comparable to MD2 control diet mice. This suggests that the kidney weight gain was pathological, and not attributed to normal adaptive growth stimulated by diet. A characteristic lesion associated with obesity is glomerulomegaly and glomerulosclerosis, manifesting as increased deposition of connective tissue matrix, cellular proliferation and leucocytic infiltration 2, 10, 36. Our histological analyses of renal tissue from mice on HFD or with increased circulating PA are consistent with this type of tissue remodelling. We have shown increased deposition of collagen and glycogen as well as increased macrophage infiltration. These pathological tissue changes are not evident in the MD2 knockout mice or mice with pharmacological MD2 inhibition.

A crucial component of the renal inflammatory injury is leucocytic extravasation and infiltration into the renal tissue sites. Macrophage infiltration, as indicated by increased CD68 localization in renal tissue, occurred in mice fed a HFD or challenged with PA. Infiltrated macrophages that take up residence in the renal tissue are likely to be highly activated, secreting pro‐inflammatory cytokines, chemoattractants, growth factors and damaging oxidants. This in turn may create a vicious positive feedback system to recruit other leucocytes and produce more pro‐inflammatory molecules. Our results suggest that, at least, renal tubular cells, mesangial cells and macrophages could contribute significantly to this positive feedback cycle of inflammation in renal tissue. We observed that macrophages adhered to PA‐stimulated tubular cells and mesangial cells in culture. Activated tubular or mesangial cells may provide a recruiting platform for macrophages into kidney tissue sites. In addition, we found that all of three cell types were potently activated by PA to produce cytokines, chemokines, and induce adhesion molecules. We believe that this may create a MD2‐dependent locus of inflammatory activity. In addition, blockade of TLR4 signalling should improve insulin sensitivity in HFD‐treated mice. Thus, MD2 blockage may also improve the insulin sensitivity here. Although the protection of MD2 blockage against renal injury is accompanied with the inhibition of inflammation in mice, it is difficult to exclude the effects of improved insulin sensitivity on renal protection. In order to ascribe the renal protection to inflammatory inhibition, we used the in vitro study in renal cells (NRK‐52E and SV40), in which, MD2 inhibition directly blocked the inflammation induced by PA stimulation. We think that the anti‐inflammatory effect of TLR4/MD2 blockage may be the most upstream mechanism for the phenotypic protections in HFD‐fed mice, including renal injury in HFD mice. It is also possible that the improvement on insulin sensitivity by TLR4/MD2 blockage also results from the inflammatory decrease in HFD‐fed mice. Also, it is interesting that the triglycerides are not elevated in MD2 knockout mice on HFD (Fig. S2). This prompts the question whether lowering triglycerides is the mechanism of renoprotective action of MD blockage. However, we do not know the mechanism yet. Such notes will be further studied by us in the future.

Our experimental elevation of the circulating PA, a predominant SFA, in mice resulted in inflammatory renal injury mimicking that produced by HFD. We acknowledge that it will be better to measure saturated fatty acids or PA in mouse kidney tissues. However, we did not find any commercial kits for the examination of PA level. The levels of FFA in control kidney and obese kidney were shown in the Figure S10, which indicated that FFA level in HFD mouse kidney was significantly higher than that in control mouse kidney. MD2 knockout mice were protected against development of PA‐induced renal injury. These finding have two important implications: i) PA is likely a predominant SFA responsible for the HFD‐associated renal inflammatory injury; and ii) the mechanism(s) by which PA induces the pathological changes were mediated mostly through MD2. We suspect that an upstream initiating event stimulated by PA is the formation of the MD2‐TLR4 complex. However, several questions remain unanswered and should be the focus of future studies. For example, the elucidation of direct cellular targets of PA in kidney tissue. In addition, understanding inflammatory stress‐induced cellular activities that regulate the progression towards renal injury would be crucial. Studies have shown that many, if not all, cell types of kidney tissue are responsive to direct stimulation by SFAs. These cells include macrophages 14, mesangial cells 7, vascular endothelial cells 20, podocytes 37 and smooth muscle cells 13. Future studies are needed to unravel this complex but important pathogenic mechanism of obesity‐associated renal injury.

In summary, our results confirm that HFD induces an inflammatory state of renal tissue, with progression towards renal tissue remodelling and functional deficits. PA, the most predominant circulating SFA, is the likely SFA responsible for the renal tissue inflammation, tissue remodelling, and dysfunction observed in HFD‐fed mice. This notion is supported by PA‐challenge studies which recapitulated HFD‐associate phenotype. Importantly, we conclude that MD2 is an important mediator of obesity‐ and SFA‐induced activation of pro‐inflammatory signalling pathways MAPKs/NF‐κB and the generation of inflammatory molecules.

## Author contributions

G.L., Q.F. and Y.W. contributed to experimental design. Q.F., L.W., X.C., D.Y., Y.Z. and P.Z. data collection. J.W. and Y.W. analysed the date and reviewed the article. H.L., G.L. and Y.W. wrote the article.

## Conflicts of interest

The authors confirm that there are no conflicts of interest.

## Supporting information


**Data S1** Materials and methods.
**Figure S1** MD2 expression and activation in the kidney tissues of high fat diet (HFD)‐fed mices
**Figure S2** MD2 knockout affects serum TG, but not LDL and TCH, in HFD‐fed mice.
**Figure S3** The quantitative data for the staining images in Figure 1G.
**Figure S4** Upper panel: an amplified image (400X) for TNF‐α staining in Figure 2J.
**Figure S5** MD2 expression and activation in the kidney tissues of mice with 2‐month HFD feeding.
**Figure S6** Administration with MD2 inhibitor L6H21 did not affect serum lipid profile in HFD‐fed mice.
**Figure S7** The quantitative data for the staining images in Figure 3D.
**Figure S8** MD2 inhibition by L6H21 prevents macrophage infiltration in HFD kidney.
**Figure S9** MD2 inhibition by L6H21 prevents high fat diet‐induced MCP‐1 expression in mouse kidney.
**Figure S10** HFD increases FFA levels in mouse kidney tissues.
**Figure S11** Palmitic acid injection increases kidney weight in mice.
**Figure S12** The quantitative data for the staining images in Figure 4C.
**Figure S13** Palmitic acid induces MD2‐dependent renal tissue fibrosis and inflammation *in vivo*.
**Figure S14** The quantitative data for the staining images in Figure S6H
**Figure S15** PA activates MD2‐dependent inflammatory activity in renal mesangial cells.
**Table S1** Primers used for real‐time qPCR assay.
