# Supplementary material for: Blockade of myeloid differentiation protein 2 prevents obesity‐induced inflammation and nephropathy
Source: J Cell Mol Med. 2017 Aug 2;21(12):3776–86. doi: 10.1111/jcmm.13287 (PMC5706499; doi:10.1111/jcmm.13287)
Supplement: Supplementary file 1 — Data S1 Materials and methods. Figure S1 MD2 expression and activation in the kidney tissues of high fat diet (HFD)‐fed mices Figure S2 MD2 knockout affects serum TG, but not LDL and TCH, in HFD‐fed mice. Figure S3 The quantitative data for the staining images in Figure 1G. Figure S4 Upper panel: an amplified image (400X) for TNF‐α staining in Figure 2J. Figure S5 MD2 expression and activation in the kidney tissues of mice with 2‐month HFD feeding. Figure S6 Administration with MD2 inhibitor L6H21 did not affect serum lipid profile in HFD‐fed mice. Figure S7 The quantitative data for the staining images in Figure 3D. Figure S8 MD2 inhibition by L6H21 prevents macrophage infiltration in HFD kidney. Figure S9 MD2 inhibition by L6H21 prevents high fat diet‐induced MCP‐1 expression in mouse kidney. Figure S10 HFD increases FFA levels in mouse kidney tissues. Figure S11 Palmitic acid injection increases kidney weight in mice. Figure S12 The quantitative data for the staining images in Figure 4C. Figure S13 Palmitic acid induces MD2‐dependent renal tissue fibrosis and inflammation in vivo. Figure S14 The quantitative data for the staining images in Figure S6H Figure S15 PA activates MD2‐dependent inflammatory activity in renal mesangial cells. Table S1 Primers used for real‐time qPCR assay. [file JCMM-21-3776-s001.doc]

**SUPPLEMENTAL MATERIAL**

**Blockade of myeloid differentiation protein 2 prevents obesity-induced inflammation and nephropathy**

Qilu Fang^1,2,#^, Lintao Wang^1,#^, Daona Yang^2,#^, Xiong Chen^3^, Yali Zhang^1^, Hazel Lum^1^, Jingying Wang^1^, Peng Zhong^1^, Xiaokun Li^1^, Guang Liang^1^,*, Yi Wang^1,2^,*

*^1^ Chemical Biology Research Center, School of Pharmaceutical Sciences, Wenzhou Medical University, Wenzhou, Zhejiang, China;*

*^2^ Affiliated Cangnan Hospital of Wenzhou Medical University, Wenzhou, Zhejiang, China*

*^3^ Department of Endocrinology, the Affiliated First Hospital, Wenzhou Medical University, Wenzhou, Zhejiang, China;*

**Methods in details, Supplementary Tables, Figures, and Legends**

**Materials and methods**

Palmitic acid, curcumin, TLR2 agonist Pam3Csk4, and bovine serum albumin (BSA) were purchased from Sigma-Aldrich (St. Louis, MO. MD2 neutralizing antibody (anti-MD2) was purchased from InvivoGen (San Diego, CA). L6H21 and curcumin were dissolved in DMSO for *in vitro* experiments and in 1% sodium carboxyl methyl cellulose (CMC-Na) for *in vivo* experiments. Antibodies for GAPDH, p38, p-p38, JNK, and p-JNK were purchased from Cell Signaling (Danvers, MA). Antibodies for IκB-α, p- IκB-α, MD2, ERK, p-ERK, TGF-β1, Collagen IV, NF-κB p65, VCAM-1, Lamin B, CD68, MCP-1, TLR4, and CD68 were purchased from Santa Cruz Biotechnology (Santa Cruz, CA). Secondary antibodies were also obtained from Santa Cruz. Mesangial cells (SV40 MES 13, ATCC-CRL-1927) and tubular epithelial cells (NRK-52E, ATCC-CRL-1571) were obtained from American Type Culture Collection (ATCC, Manassas, VA, USA). The cells were maintained at 37°C under a humidified 5% CO2 in Dulbecco’s modified Eagle’s medium (DMEM) (Gibcol, Eggenstein, Germany) containing 5.5 mM D-glucose (low glucose, LG) supplemented with 10% FBS (Gibco), 100 U/mL penicillin and 100 U/mL streptomycin.

**Ultrasound kidney hemodynamic function**

Kidney hemodynamic function was evaluated by measures of blood flow velocity and systolic to diastolic pressure ratio of the left renal artery. Mice were anesthetized and placed in the supine position on a heating pad to maintain body temperature at 36–37 °C. Ultrasound kidney function was assessed using a Vevo 770 high-resolution imaging system (Visual Sonics, Canada) equipped with a high-frequency ultrasound probe (RMV-707B). Hair was removed and aquasonic clear ultrasound gel (Parker Laboratories, Fairfield, NJ) was applied to the lower back to optimize visualization of the renal artery.

**Measurement of circulating lipids and renal function markers**

Total triglycerides (TG), low-density lipoproteins (LDL), and total cholesterol (TCH) were measured in serum samples using commercial kits (Nanjing Jiancheng, Jiangsu, China). Serum was also used to measure blood urea nitrogen (BUN) and creatinine. BUN, creatinine, albumin, uric acid, and total proteins were measured in urine samples using commercial kits (Nanjing Jiancheng, Jiangsu, China)

**Cell culture and isolation**

Mouse primary macrophages (MPMs) and rat primary macrophages were prepared from peritoneal cavity of adult male C57BL/6 mice and SD rats, respectively, as described previously[1]. NRK52E rat renal proximal tubular epithelial cells and SV40 MES 13 mouse kidney mesangial cells were cultured in DMEM/F12 (Gibco/BRL life Technologies, Eggenstein, Germany) containing 4.5 g/L glucose supplemented with 10% FBS (Hyclone, Logan, UT, USA), 100 U/mL penicillin, and 100 mg/mL streptomycin.

**Histology and immunohistochemistry**

Paraformaldehyde-fixed kidney tissues were embedded in paraffin and sectioned at 5 μm thickness. Tissue sections were stained with hematoxylin and eosin (H&E), 0.5% periodic acid and Schiff solution (PAS), and 0.1% Sirius red and Masson’s trichrome stain. Sections were also stained for CD68 and TNF-α using routine methods. Briefly, sections were treated with 3% H_2_O_2_ for 10 min, blocked with 1% BSA for 30 min, and incubated with primary antibodies overnight at 4 °C. Horseradish peroxidase (HRP)-conjugated secondary antibody was added at 1:400 for 1 h. The reaction was visualized with DAB solution. Slides were counterstained with hematoxylin and mounted.

**Macrophage adhesion assay**

NRK52E or SV40 cells were pretreated with L6H21 for 1 h and then stimulated with 100 µM PA for 24 h. Taking NRK52E as an example, rat primary macrophages (mouse primary macrophages for SV40 cells) were added onto the blank control (no NRK52E cells, DMSO group), non-treated NRK52E (Mac group), L6H21 alone-treated NRK52E (Mac+621 group), PA-treated NRK52E (Mac+PA group), or PA+L6H21-treated NRK52E (Mac+PA+621 group), respectively, and incubated for 15 min. After washing by PBS, cells in four groups were then stained for CD68 using PE-conjugated secondary antibody; nuclei of cells were stained and indicated by DAPI. Images were captured using a Nikon epifluorescence microscope equipped with a digital camera.

**Determination of cytokine levels**

The levels of TNF-α, IL-6 and MCP-1 in cell culture media and homogenized kidney tissues were determined using ELISA kits (Bioscience, San Diego, CA). Cytokine levels were normalized to total protein of the cell pellet or tissue accordingly.

**Western immunoblot analysis**

Lysates were prepared from cells or kidney tissues and subjected to routine Western immunoblot analysis with the respective primary antibodies and HRP-conjugated secondary antibody. Bands were visualized using enhanced chemiluminescence reagents (Bio-Rad, Hercules, CA). Band density was quantified by Image J analysis version 1.38e (NIH) and normalized to their respective controls.

For co-immunoprecipitation, cell and tissue lysates were immunoprecipitated with anti-MD2 antibody overnight in 4 °C. Protein A/G beads were added and samples were shaken at room temperature for 2 h. Proteins were evaluated by Western blot analysis.

**Real-time quantitative PCR**

Total RNA was isolated from cells or tissues using TRIZOL (Thermo Fisher, Carlsbad, CA). Reverse transcription and quantitative PCR were performed using M-MLV Platinum RT-qPCR Kit (Thermo Fisher). Real-time qPCR was carried out in Eppendorf Realplex 4 (Eppendorf, Hamburg, Germany). Primers for genes (Supplementary Table 1) were obtained from Thermo Fisher (Shanghai, China). The relative amount of each gene was normalized to β-actin.

**Electrophoretic mobility shift assay (EMSA)**

Nuclear extracts from cultured cells were prepared using the Nuclear and Cytoplasmic Protein Extraction Kit and protein concentrations were determined. NF-κB and AP-1 activities were determined using EMSA. After electrophoresis, DNA-protein complex was transferred to a nylon membrane and cross-linked. The biotinylated-labelled DNA was detected by chemiluminescence.

**Table S1: Primers used for real-time qPCR assay**

| **Gene** | **species** | **FW** | **RW** |
| --- | --- | --- | --- |
| IL-6 | mouse | GAGGATACCACTCCCAACAGACC | AAGTGCATCATCGTTGTTCATACA |
| IL-33 | mouse | GATGGGAAGAAGGTGATGGTG | TTGTGAAGGACGAAGAAGGC |
| IL-1β | mouse | GGAAGCACGGCAGCAGAATA | CCATCAGAGGCAAGGAGGAA |
| TNF-α | mouse | TGATCCGCGACGTGGAA | ACCGCCTGGAGTTCTGGAA |
| IFN-γ | mouse | GCCAAGTTTGAGGTCAACAAC | ATCAGCAGCGACTCCTTTTC |
| ICAM-1 | mouse | GCCTTGGTAGAGGTGACTGAG | GACCGGAGCTGAAAAGTTGTA |
| VCAM-1 | mouse | TGCCGAGCTAAATTACACATTG | CCTTGTGGAGGGATGTACAGA |
| Collagen1 | mouse | TGGCCTTGGAGGAAACTTTG | CTTGGAAACCTTGTGGACCAG |
| Collagen4 | mouse | ATCTCTCAGGGTGCGTGC | CTCATTGATACCACAAATGCA |
| TGF-β1 | mouse | TGACGTCACTGGAGTTGTACGG | GGTTCATGTCATGGATGGTGC |
| β-Actin | mouse | CCGTGAAAAGATGACCCAGA | TACGACCAGAGGCATACAG |
| MCP-1 | mouse | TCACCTGCTGCTACTCATTCACCA | TACAGCTTCTTTGGGACACCTGCT |

**
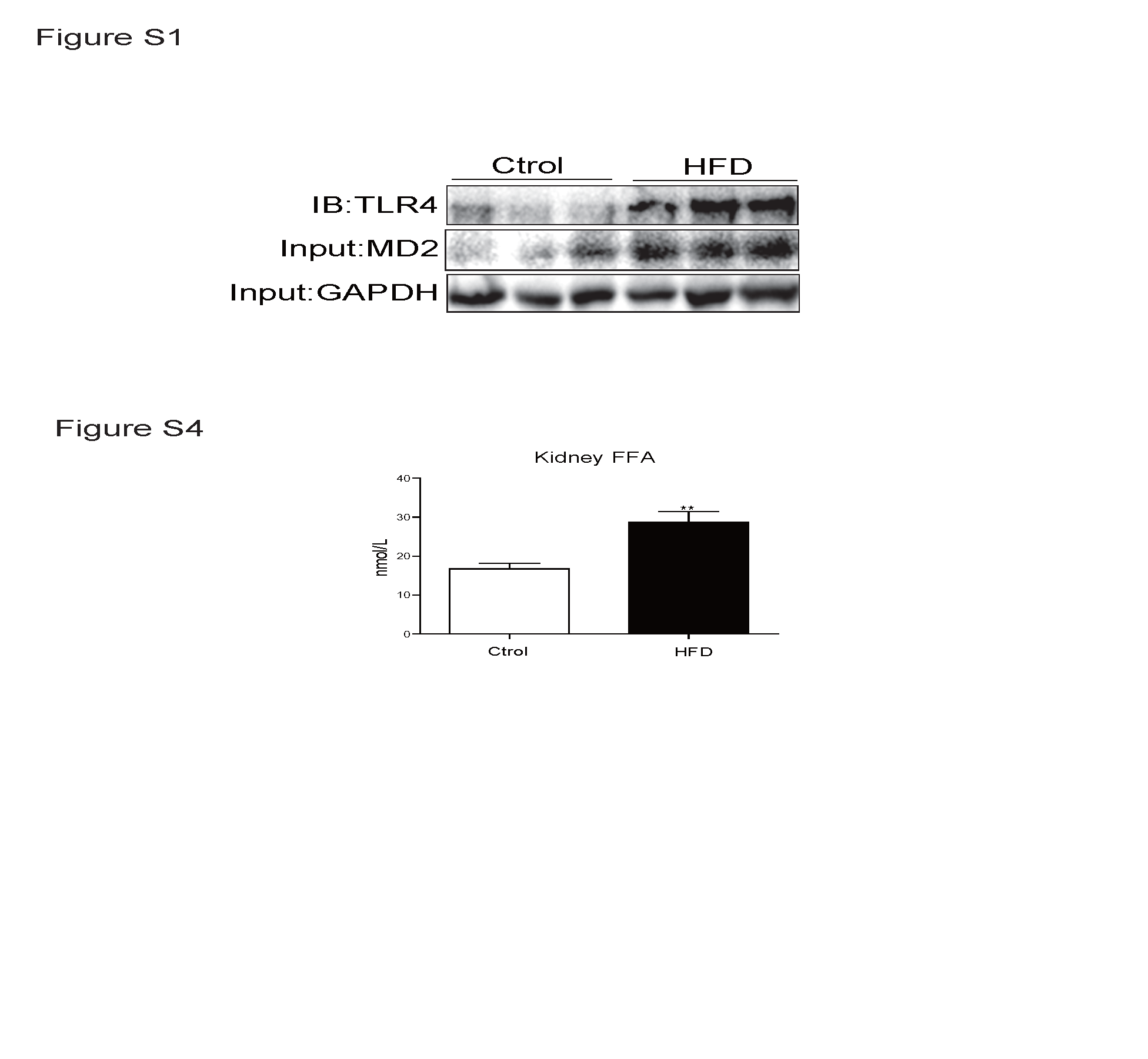
**

**Supplement Figure S1. MD2 expression and activation in the kidney tissues of high fat diet (HFD)-fed mice.**

C57BL/6 mice were fed a high fat diet for 4 months. Controls were fed standard chow. Total proteins from kidney samples were subjected to co-immunoprecipation. Proteins samples used for this figure were derived from our previously study[2].

**
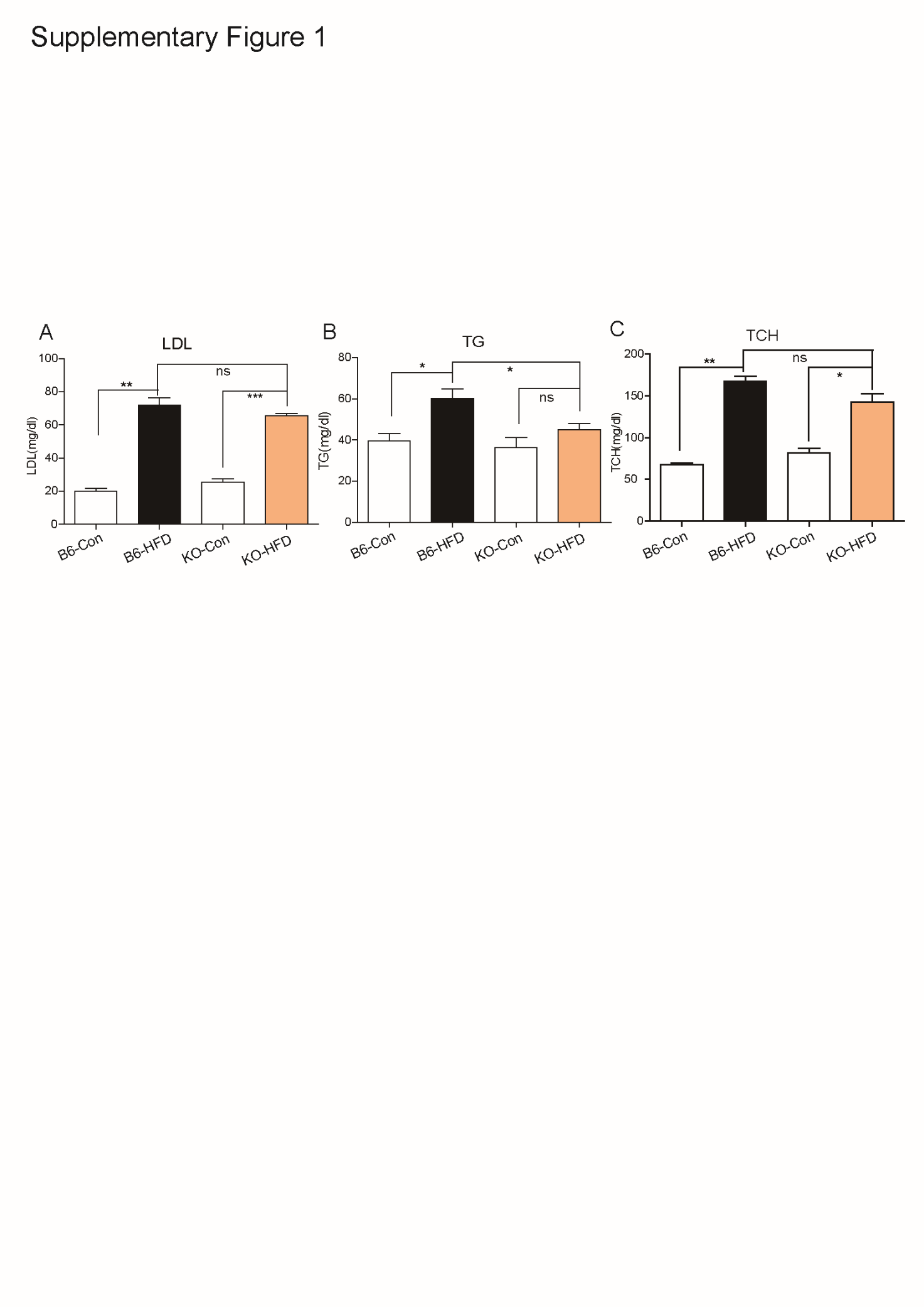
**

**Supplementary Figure S2. MD2 knockout affects serum TG, but not LDL and TCH, in HFD-fed mice.**

Wild-type (B6) or MD2^-/-^ (KO) mice were fed a HFD or normal control diet (Ctrol) for 4 months. Blood was collected for evaluation at time of sacrifice. Graphs showing **A**) serum low-density lipoprotein (LDL), **B**) serum triglycerides (TG), and **C**) serum total cholesterol (TCH). Values are reported as means ± SEM; n=5-7; **P*<0.05, ***P*<0.01, ****P* <0.001, ns, not significant.

**
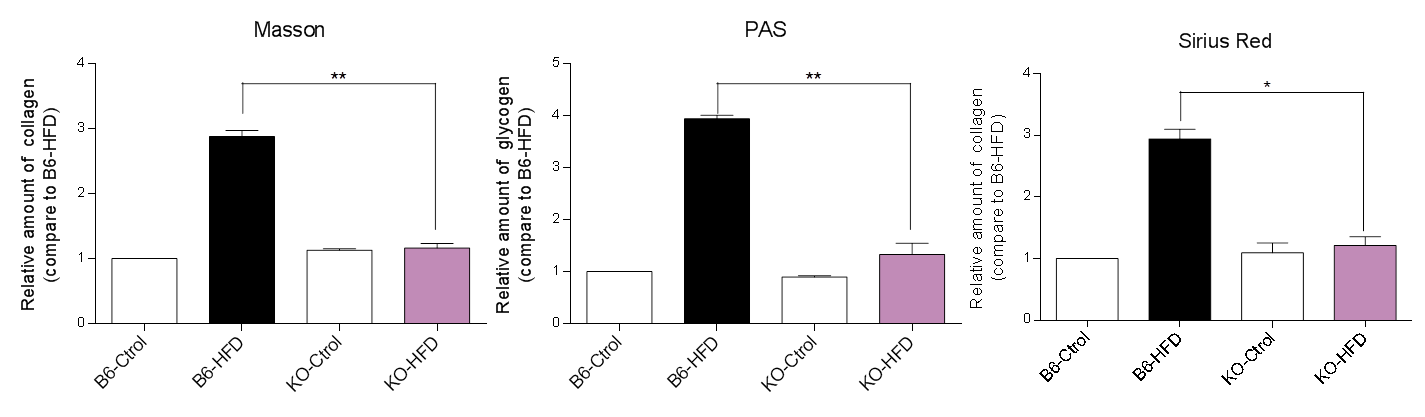
**

**Supplement Figure S3.** The quantitative data for the staining images in Figure 1G.


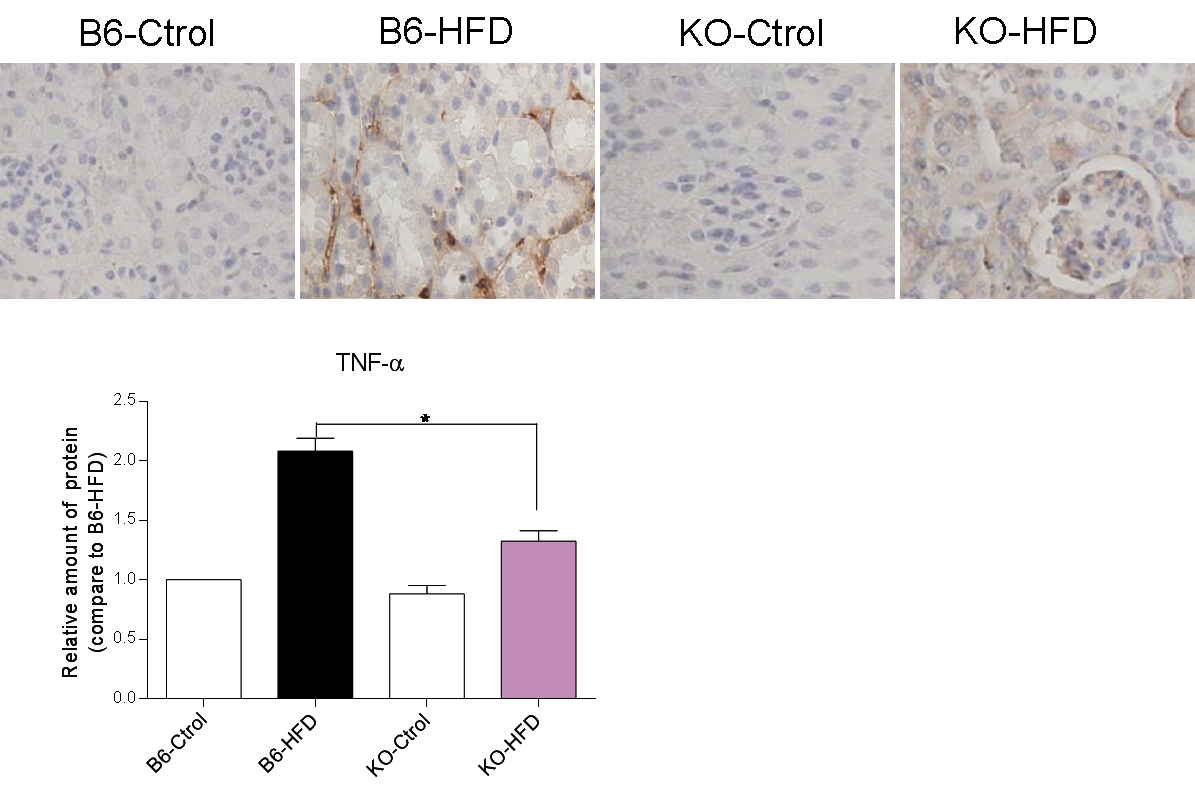


**Supplement Figure S4.** Upper panel: an amplified image (400X) for TNF-α staining in Figure 2J. Below panel: the quantitative data for the staining images in Figure 2J.


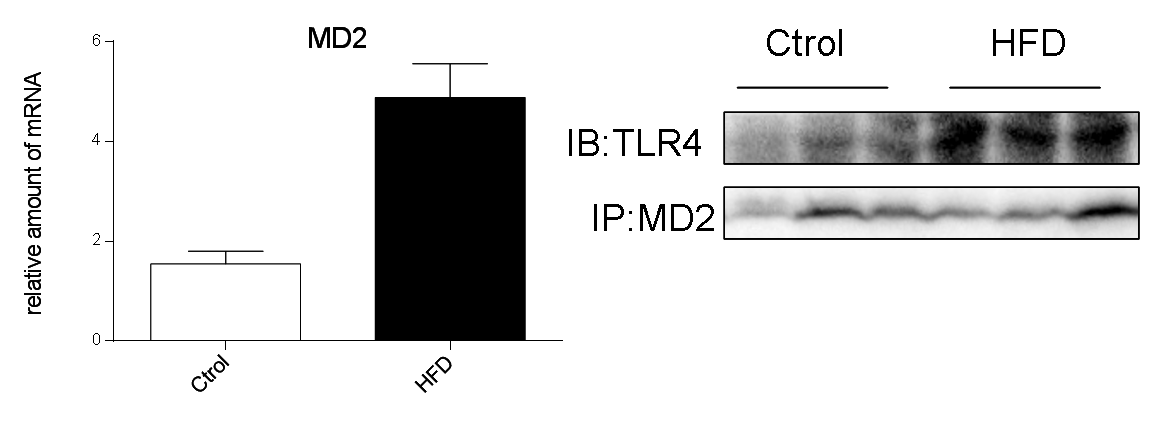


**Supplement Figure S5. MD2 expression and activation in the kidney tissues of mice with 2-month HFD feeding.**

C57BL/6 mice were fed a high fat diet for 2 months. Controls were fed standard chow. Right panel: the mRNA level of MD2 was examined by real-time qPCR assay. Left panel: total proteins from kidney samples were subjected to co-immunoprecipation.

**
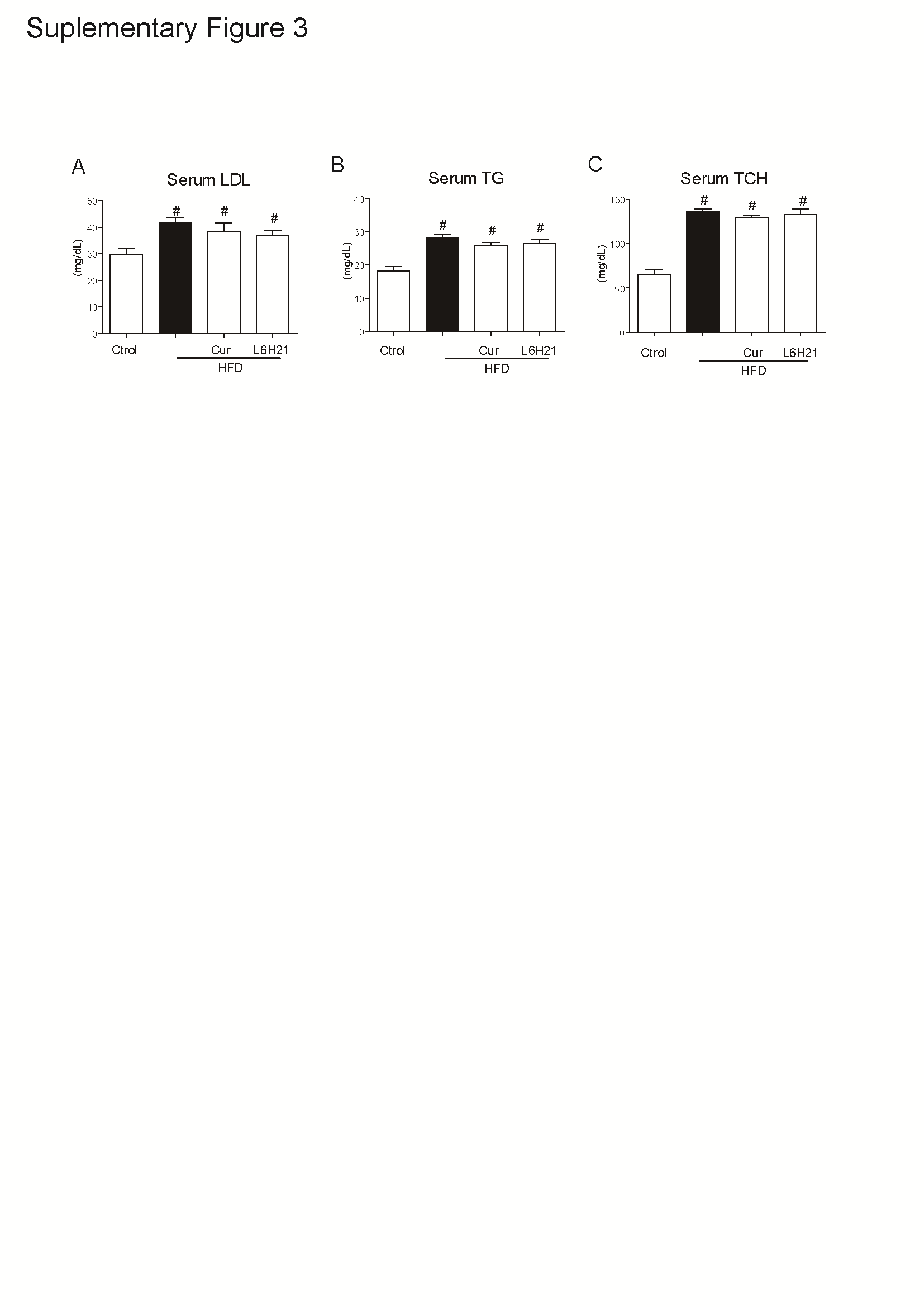
**

**Supplementary Figure S6. Administration with MD2 inhibitor L6H21 did not affect serum lipid profile in HFD-fed mice.**

C57BL/6 mice were fed a high fat diet (HFD) or normal diet (ctrol) for 2 months, and were then given 20 mg/kg L6H21 or 50 mg/kg curcumin (cur) every 2 days for 2 months by oral gavage. Graphs showing **A**) serum low-density lipoprotein (LDL), **B**) serum triglycerides (TG), and **C**) serum total cholesterol (TCH). Values are reported as means ± SEM; n=5-7; #*P*<0.05, versus Ctrol.


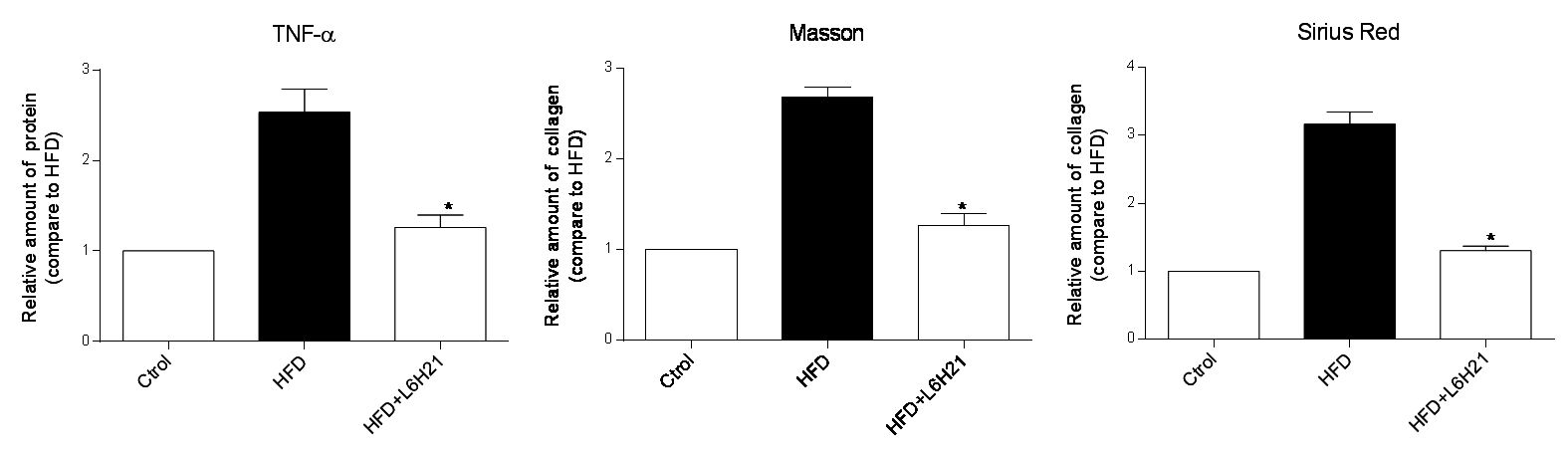


**Supplement Figure S7.** The quantitative data for the staining images in Figure 3D.


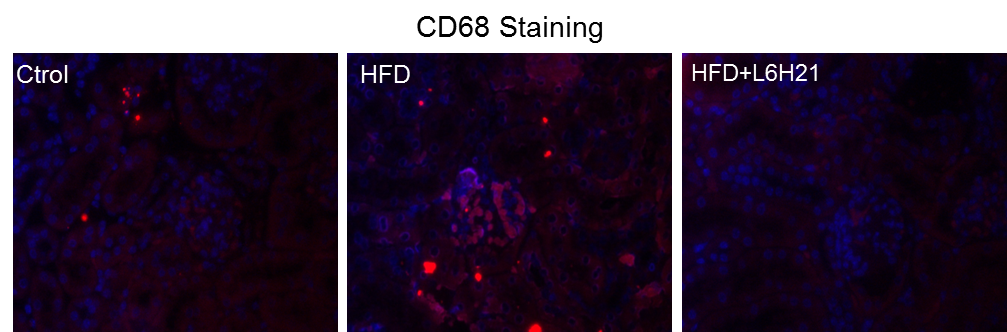


**Supplementary Figure S8. MD2 inhibition by L6H21 prevents macrophage infiltration in HFD kidney.** C57BL/6 mice were fed a HFD or control diet (ctrol) for 2 months. Mice were then treated with L6H21 every 2 days for 2 months and kidney tissues were evaluated. Representative microscopic images of [immunofluorescence staining](http://www.baidu.com/link?url=iZWHi4qWjAEYY6AyntlZ2Z3eQ2tw82udmWCQq2KKOss096JtNXuwCiaBHaXZENPJFhRSNkyu8LXUHbjJOh2KFVJ8qVQ5nMSwQ2vdmmQGObnHb1vE1ZKCDZIAbqEboBUd) for CD68 (red color) in kidney sections.

**Supplementary Figure S9. MD2 inhibition by L6H21 prevents high fat diet-induced MCP-1 expression in mouse kidney.**

C57BL/6 mice were fed a HFD or control diet (ctrol) for 2 months. Mice were then treated with L6H21 every 2 days for 2 months and kidney tissues were evaluated. Real-time qPCR analysis showing MCP-1 mRNA levels in kidney tissues of mice in three groups. [mRNA levels normalized to β-actin; means ± SEM; n=7/8 in four groups; ****P*<0.001 versus HFD].


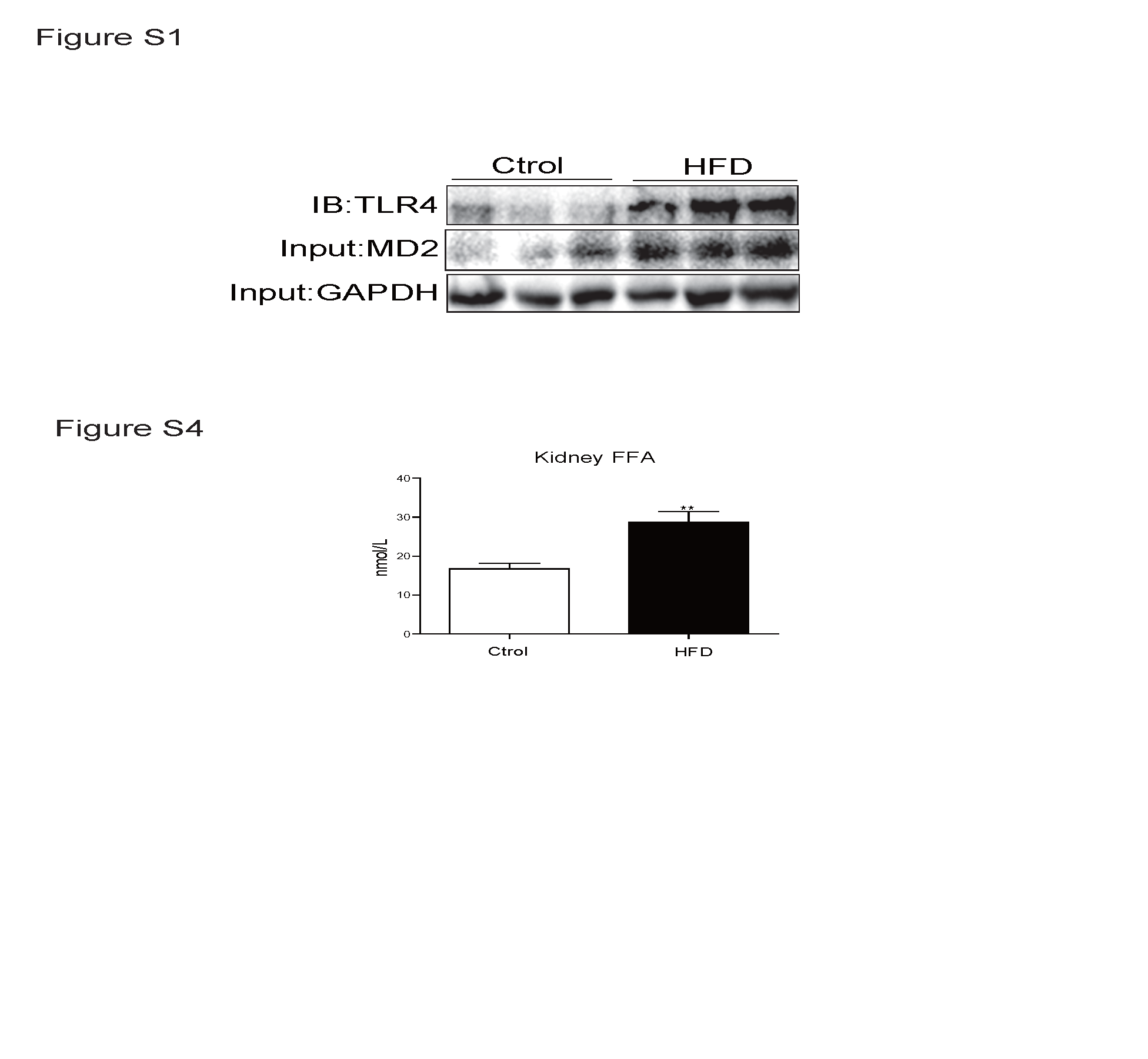


**Supplementary Figure S10. HFD increases FFA levels in mouse kidney tissues.** C57BL/6 mice were fed a high fat diet (HFD) or normal diet (ctrol) for 4 months, and kidneys were harvested for evaluation. Tissues were homogenized and FFA levels were measured using ELISA kit (Catalog #ml002083; MlBio Technology, Shanghai, China). Values are reported as means ± SEM; n=8; ***P*<0.01.


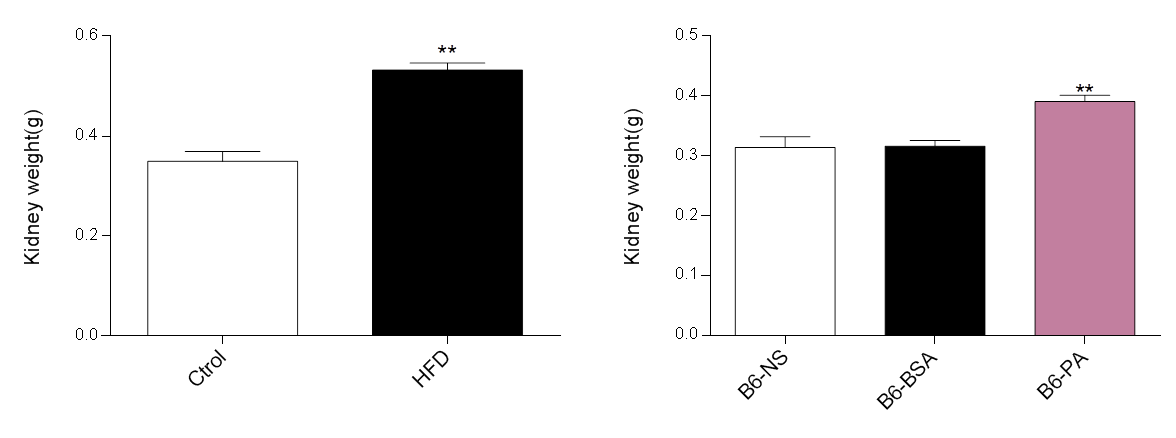


**Supplementary Figure S11. Palmitic acid injection increases kidney weight in mice*.*** Wildtype (B6) mice were challenged with palmitic acid (PA) in BSA solution twice daily for 2 weeks. Non-treated mice were used as a blank control and BSA-injected mice was used as a vehicle control. When sacrifice, the kidneys in three groups were collected and weighted. ** P<0.01, v.s. B6-NS group.


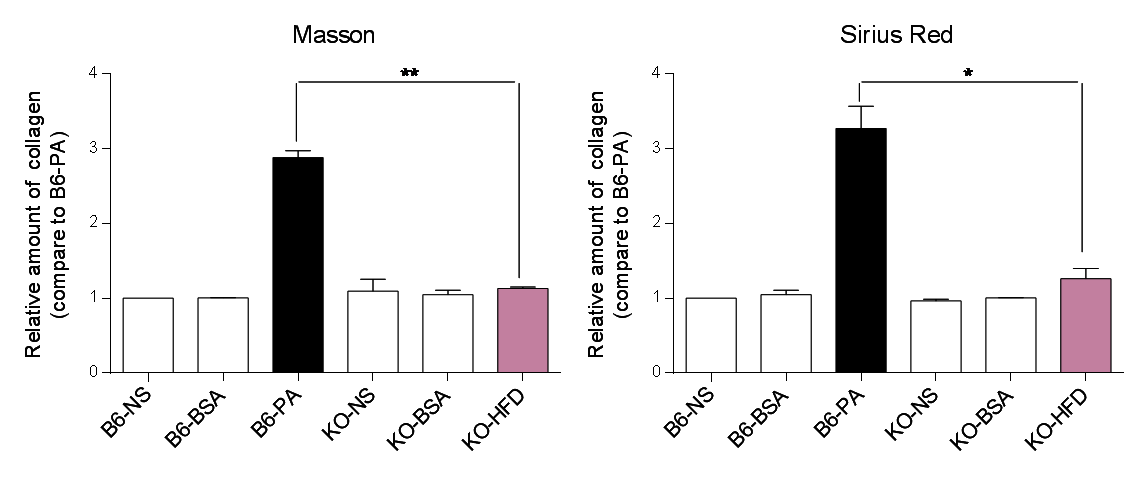


**Supplement Figure S12.** The quantitative data for the staining images in Figure 4C.

**
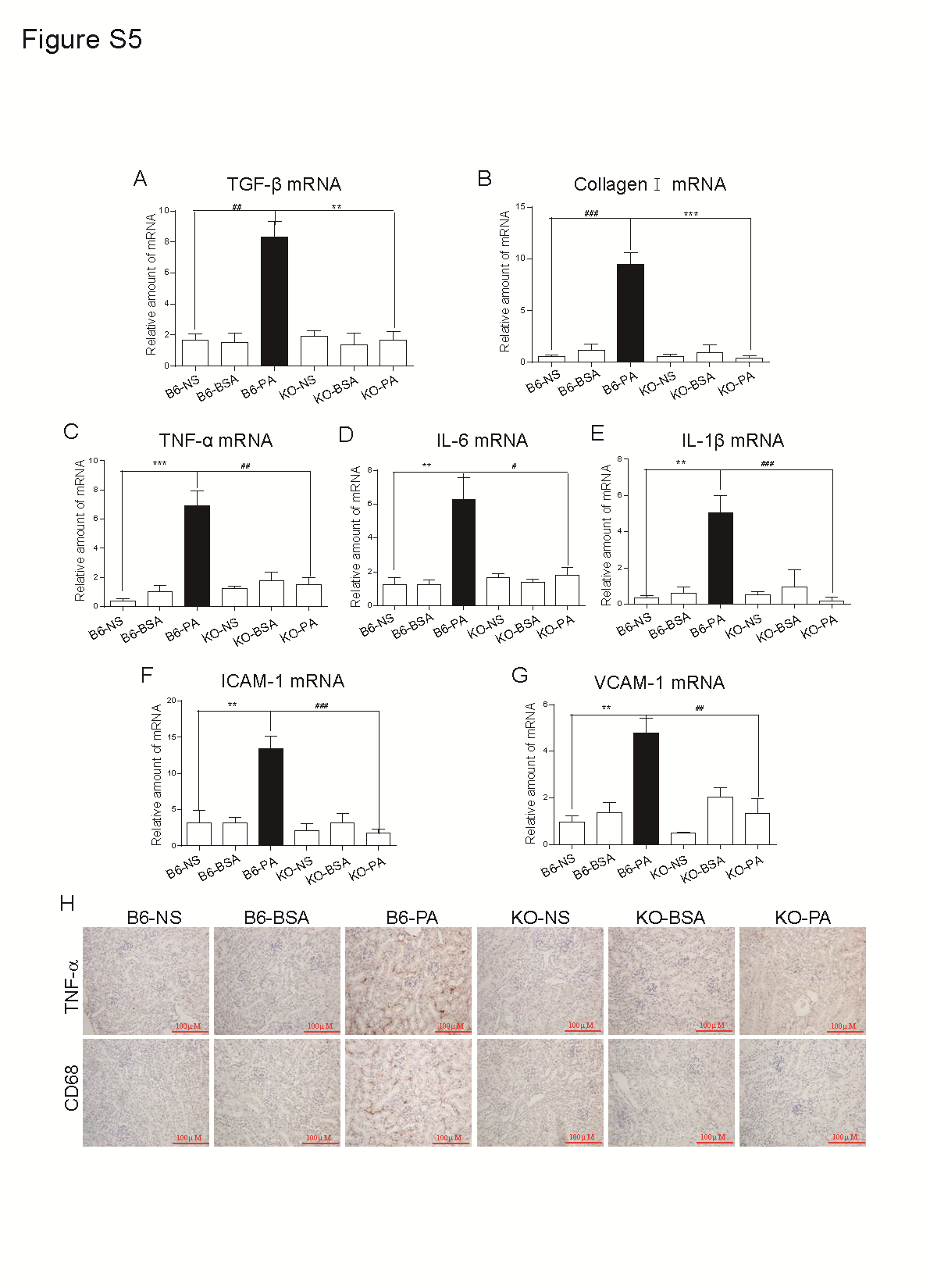
**

**Supplementary Figure S13. Palmitic acid induces MD2-dependent renal tissue fibrosis and inflammation *in vivo.*** (**A**-**B**) mRNA levels of TGF-β1 (**A**) and Collagen I (**B**) in mouse kidneys. [mRNA levels normalized to β-actin; mean ± SEM; n=8; ***P*<0.01, and ****P*<0.001, KO-PA versus B6-PA; ^##^*P* <0.01, and ^###^*P* <0.001, B6-NS versus B6-PA]. (**C**-**G**) mRNA levels of inflammatory cytokines and adhesion molecules in kidney samples showing TNF-α (**C**), IL-6 (**D**), IL-1β (**E**), ICAM-1 (**F**), and VCAM-1 (**G**). [mRNA levels normalized to β-actin; mean ± SEM; n=5/8; ***P*<0.01, and ****P*<0.001, B6-NS versus B6-PA; ^#^*P* <0.05, ^##^*P* <0.01, and ^###^*P* <0.001, KO-PA versus B6-PA]. (**H**) Representative immunohistochemical detection of TNF-α and CD68 in kidney tissue sections. The quantitative data were shown in the supplementary Fig. S14.


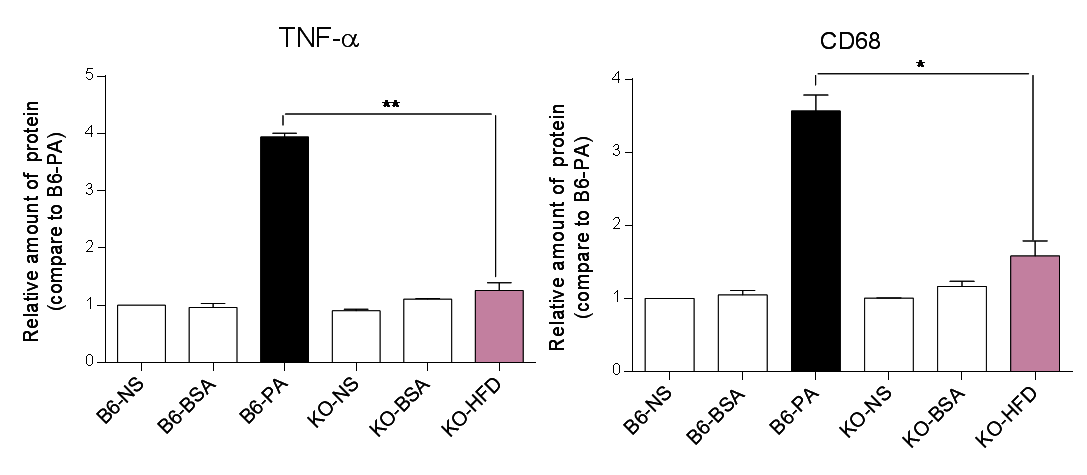


**Supplement Figure S14.** The quantitative data for the staining images in Figure S6H.

**Supplementary Figure S15. PA activates MD2-dependent inflammatory activity in renal mesangial cells.** Mouse SV40 mesangial cells (SV40) were treated with 100 µM PA at indicated times with or without 1 h pretreatment with L6H21 (2.5-10 µM) or curcumin (10 µM). **A**) Representative Western blot analysis of IκB-α, p-ERK, p-JNK, and p-p38 in SV40 cells stimulated with PA for 30 min (GAPDH served as loading control). **B**) mRNA levels of TNF-α, IL-6, and IL-1β in SV40 cells stimulated with PA for 6 h. **C**) mRNA expression of adhesion molecules ICAM-1 and VCAM-1, and chemokine MCP-1 in SV40 cells stimulated with PA for 6 h. For panels B and C, mRNA levels were normalized to β-actin [means ± SEM; n=3; **P*<0.05, ***P*<0.01, and ****P*<0.001 versus PA]. **D**) Representative Western blot of MCP-1 and VCAM-1 expression in SV40 mesangial cells stimulated with PA for 12 h. **E**) Adhesion assay was conducted by stimulating SV40 cells with 100 μM PA for 24 h with or without 1 h pretreatment with L6H21. Mouse primary macrophages (Mac) were then overlaid for 15 min. Macrophage adhesion on the surface of SV40 cells was detected by CD68 immunostaining; nuclei of cells were indicated by DAPI staining [representative fluorescence image of macrophage adhesion shown (Orange); 3 separate determinations].

**References**

[1] Pan Y, Wang Y, Zhao Y, Peng K, Li W, Wang Y, et al. Inhibition of JNK phosphorylation by a novel curcumin analog prevents high glucose-induced inflammation and apoptosis in cardiomyocytes and the development of diabetic cardiomyopathy. Diabetes. 2014:DB_131577.

[2] Fang Q, Deng L, Wang L, Zhang Y, Weng Q, Yin H, et al. Inhibition of Mitogen-Activated Protein Kinases/Nuclear Factor kappaB-Dependent Inflammation by a Novel Chalcone Protects the Kidney from High Fat Diet-Induced Injuries in Mice. The Journal of pharmacology and experimental therapeutics. 2015;355:235-46.
